# Editorial: TGF-β signalling pathways and their enigmatic role as a friend and foe in human health and diseases

**DOI:** 10.3389/fmolb.2023.1232454

**Published:** 2023-06-20

**Authors:** Venkaiah Betapudi, Birija Sankar Patro, Shizuya Saika, Shaik O. Rahaman

**Affiliations:** ^1^ Department of Health and Human Services, Washington, DC, United States; ^2^ Bio-Organic Division, Bhabha Atomic Research Centre, Mumbai, India; ^3^ Wakayama Medical University Hospital, Wakayama, Japan; ^4^ University of Maryland, College Park, MD, United States

**Keywords:** TGF-β, signalling pathways, cellular processes, smad-dependent and independent signalling pathways, human health and diseases

Our development from a single embryonic cell to an adult body consisting of ∼50 trillion cells relies on an intricate network of signalling pathways. These pathways, modulated by both physical and chemical cues, coordinate the actions of distant organs, tissues, and cells, thereby fine-tuning their functions. Among these modulators, Transforming growth factor beta (TGF-β) plays a crucial role in regulating cell growth, differentiation, proliferation, migration, apoptosis, and homeostasis. Existing as TGF-β1, TGF-β2, and TGF-β3, it activates both SMAD-dependent and independent signalling pathways. However, disruptions in these pathways can have diverse impacts on human health and disease development. Our limited understanding of TGF-β′s functions presents a significant obstacle in developing new and comprehensive treatment options.

In this Research theme, eminent researchers in the discipline tackle the range of TGF- β functions, examining its effects on other signal transduction pathways, cellular functions, disease progression, and potential treatment of diseases.

Abdelhamid et al. examined the role of microRNAs and the TGF-β/SMAD pathways in transforming dormant hepatic stellate cells (HSCs) into proinflammatory, contractile, and fibrogenic myofibroblasts, sparking fibrogenesis and liver fibrosis, a condition responsible for nearly two million global deaths annually due to limited therapeutic options. They treated mice with liver fibrosis using piperine, an alkaloid found in black pepper, observing a restoration of SMAD-7 expression, a notable decrease in microRNA-17 expression and TGF-β/SMAD pathway activity, as well as suppression of activated HSCs and collagen deposition in hepatocytes. Their research indicates that piperine, could potentially serve as a promising treatment for liver fibrosis by inhibiting the TGF-β/SMAD pathway.

Osteoporosis, a condition causing bone fragility due to an imbalance between bone-forming and bone-absorbing osteoclasts, affects over 200 million people worldwide. TGF-β/SMAD signalling pathways play crucial roles in regulating osteoblastic and osteoclastic differentiation during skeletal development, bone formation, and bone homeostasis, signifying a significant link with bone remodelling. Thus, these pathways present potential targets for novel osteoporosis treatments. In a comprehensive review, Zou et al. highlighted the latest developments in this area, outlining the role of TGF-β/SMAD pathways in bone remodelling. Their study illuminates potential avenues for developing treatment strategies for osteoporosis, further advancing our understanding of this complex disease.

Alström Syndrome (ALMS) is a rare genetic disease presenting progressive vision and hearing loss, cardiomyopathy, obesity, type 2 diabetes, fibrosis, kidney failure, and short stature. With approximately 1,200 identified cases globally and no current treatment, it remains a significant health challenge. ALMS results from mutations in the ALMS1 gene, encoding a protein of 4,169 amino acids across 23 exons; the exact causative mechanisms, however, are yet to be elucidated. Bea-Mascato et al. highlighted the vital role of the ALMS1 gene in regulating TGF-β/SMAD3 signalling pathways and cellular migration and adhesion through their comprehensive methodology, encompassing molecular cell biology, gene knockout models, and proteomic analyses. This research could pave the way for the development of gene therapy-based treatment approaches for ALMS patients.

Primary Open-Angle Glaucoma (POAG) ranks as a leading cause of irreversible blindness, affecting an estimated 57 million individuals globally. At present, the treatment options primarily involve eye drops and laser trabeculoplasty, both aimed at lowering eye pressure. No novel treatments have emerged thus far. Prior research suggested the involvement of the matricellular protein CCN2/connective tissue growth factor (CTGF), abnormal TGF-β2 expression, and its antagonist bone morphogenetic proteins (BMP) in the activation of the actin cytoskeleton and in elevating intraocular pressure (IOP), a key risk factor for POAG. Nevertheless, the underlying mechanisms remain poorly understood. In a seminal study, Dillinger et al. exhibited a direct link between CCN2/CTGF and TGF-β and BMP, as well as the activation of the RhoA/ROCK and ERK signalling pathways. They utilized a transgenic mouse model system and an immortalized Human Primary Trabecular Meshwork (HTM) cell system. Their findings led to the conclusion that CCN2/CTGF operates as a regulator of the homeostatic balance between BMP and TGF-β signaling pathways, a balance disrupted in primary open-angle glaucoma.

TGF-β plays a seemingly paradoxical role in health and cancer, a disease arising from the abnormal growth, proliferation, and metastasis of normal cells. It is important to note that cancer is not a single disease but rather a collection of over 100 different diseases. The four main types of cancer are carcinoma (skin), sarcoma (connective tissue), leukemia (blood), and lymphoma (lymph nodes). The American Cancer Society reported over 10 million cancer-related deaths in 2020. Although many cancer treatments are available, a definitive cure remains elusive. Numerous studies have indicated that TGF-β signalling pathways inhibit cancer growth in its early stages but encourage tumor growth in the late stages. Treatments aimed at TGF-β synthesis, TGF-β-TGF-β receptor complexes, or TGFβ receptor kinase activity have shown promise in tissue culture and animal models, yet failed in clinical trials. Trelford et al. conducted an extensive review of the TGF-β signalling pathways, the role of TGF-β during tumorigenesis, and how protein quality control pathways contribute to the tumor-promoting consequences of TGF-β signalling. This review may serve as a foundation for further understanding and developing drug strategies for combating cancer.

Endothelial cells form a 60,000 miles-long closed loop elastic vascular system or circulatory system-arteries, veins and capillaries that carry blood and lymph fluid through the body. The fate of endothelial cells not only impacts the circulatory system, respiratory system, digestive system, kidneys and urinary system and body temperature control but also results into vascular diseases. According to Centers for Disease Control and Prevention, United States of America, an estimated 523 million people had some form of vascular diseases and attributed to approximately 19 million deaths in 2020. Chen et al. wrote an elegant review on the role of this multifaceted TGF-β in determining the fate of endothelial cells and circulatory system.

In summary, there is a global aspiration to decode the multiple factors and their synergistic effects involved in TGF-β signalling. A comprehensive understanding of their roles in diverse cellular processes could pave the way for discovering innovative therapeutic strategies for various diseases including cancer, fibrosis, Marfan syndrome, Osler-Weber-Rendu syndrome, CCM, HHT, CARASIL, osteoporosis, glaucoma, glomerulosclerosis, diabetic kidney disease, cerebrovascular disease, and many more. This research theme, enriched by insights from 32 esteemed authors across the globe, tackles each of these crucial issues. It aims to equip scientists, drug developers, and clinicians with valuable knowledge on the dual roles of TGF-β signalling pathways in human health and disease progression. Moreover, this research hopes to provide investigators an extensive overview of cutting-edge issues in drug development, further enriching their understanding of TGF-β.

